# Thermal and Rheological Performances Evaluation of a Modified Biopolymer for Fracturing Fluid System

**DOI:** 10.3390/molecules27227776

**Published:** 2022-11-11

**Authors:** Guoyan Ma, Le Wang, Chao Hao, Chunbao Du, Hongfei Ma

**Affiliations:** 1 College of Chemistry and Chemical Engineering, Xi’an Shiyou University, Xi’an 710065, China; 2 Shaanxi Key Laboratory of Continental Shale Gas Accumulation and Exploitation, Xi’an 710065, China; 3 CCDC Drilling & Production Engineering Technology Research Institute, Xi’an 710018, China; 4 Department of Chemical Engineering, Norwegian University of Science and Technology, Sem Sælands vei 4, 7034 Trondheim, Norway

**Keywords:** biopolymers, modified konjac glum, thermal stability, shear resistance, viscoelastic property

## Abstract

Developing an efficient fracturing fluid system is an enduring hot topic in the petrochemical industries, especially regarding the exploitation of limited oil. Biopolymers, especially polysaccharides (e.g., konjac gum, guar gum), are widely applied as fracturing fluids in fracturing as a result of their advantages. Herein, we propose an easy method of modifying konjac gum (KGM) using isopropanol, sodium hydroxide, and chloroacetic acid to obtain modified konjac glum (MKGM). The MKGM and KGM gels were also obtained by using the self-prepared organic titanium high-temperature stabilizer and organic borate cross-linker. The prepared MKGM was characterized by multiscale techniques, including attenuated total reflection Fourier transform infrared (ATR-FTIR), X-ray diffraction (XRD), thermal gravimetric analysis (TGA), differential scanning calorimetry (DSC), and rheology properties. The ATR-FTIR results showed that the etherification modification reaction occurred as designed. The XRD results showed that the regularity of KGM was destroyed after modification. The TGA and DSC results showed that the thermal stability improved. Rheology measurements illustrated that the temperature and shear resistance of MKGM were better than those of KGM. The MKGM gel could be applied in fracturing fluid systems at a lower frequency through viscoelastic measurements.

## 1. Introduction

Biopolymers, which are found in plants, microorganisms, and animals, are one form of polymer categorized based on their source. They have several advantages—such as homogeneous shapes and sizes, biodegradability, biocompatibility, environmental friendliness, low cost, ease of modification, and accessibility—over synthetic polymers. Polynucleotides, polypeptides, and polysaccharides are the three types of biopolymers. They all comprise bio-based monomer units, and these units can be bound together through covalent bonds to form larger bio-based polymer molecules [1].

**Figure 1:**
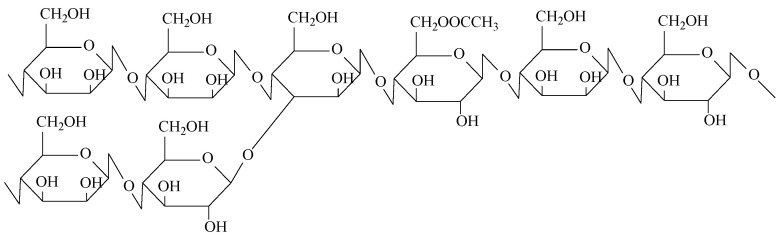
The structure of KGM.

Konjac glucomannan (KGM) is a widely used water-soluble nonionic polysaccharide with a high molecular weight. KGM is usually found in tubers of Amorphophallus konjac, which mainly grows in Asia, such as in China and Japan. It is composed of β-1,4 linked *D*-mannose and *D*-glucose residues with a reported ratio of 1.6:1 or 1.4:1. *O*-acetyl substitutions randomly attach to the C-6 position of saccharide units along the molecule, approximately 1 per 19 sugar residues, and some short branches are linked to mannoses by joint C-3 [2,3,4]. The typical structure of KGM is shown in Figure 1.

Considerable effort has been devoted to the study of KGM in China, Japan, and Europe for its wide application. The KGM solution has a high viscosity, and it could form a thermally stable gel upon the addition of alkaline cross-linkers. It has the potential to be used as a thickener, gelation or adsorbing agent, and film-former [5]. It would also be necessary to have a broader range of KGM properties available. Therefore, it would be helpful to modify KGM to alter its water absorption or other properties. There are some reports in the literature on KGM modification, including acetylation, methylation, and oxidation [6,7,8,9,10,11,12]. Cheng et al. [13] prepared partially acid-hydrolyzed konjac glucomannan. When the degree of acid hydrolysis, molecular weight, and molecular weight distribution of the polymers were controlled, the properties of edible films derived from acid-modified KGM were studied in detail. Xu et al. [14] prepared a novel thermoplastic material called thermoplastic konjac glucomannan (TKGM) through graft copolymerization in the laboratory by using KGM, vinyl acetate (VAc), and methyl acrylate (MA). The product was blended with polylactide (PLA) to improve processing and comprehensive mechanical properties. The PLA/TKGM blends were suitable for conventional molding and extrusion technologies. The research on KGM so far has been limited to the isolation and characterization of the solution and bulk properties and to the gelling behavior, mainly for food and coating applications. In addition, KGM can be extruded into fracturing fluid applications.

The main role of the fracturing fluid in reservoir stimulation is to transit pressure and induce cracks in a formation, then to carry and lay the proppants in a specified location for hydraulic cracking. Therefore, to carry sand, the fluid must have a specific viscosity. The aqueous solution and gel of guar gum [15,16], diutan gum [17], and KGM have been extensively used in hydraulic fracturing fluids. Since KGM dispersions have the highest viscosity of the 12 polysaccharides tested [4], it is important for KGM to be applied in the field of hydraulic fracturing. Hydraulic fracturing has been used in approximately 70% of fracturing fluids. However, the viscosity of KGM decreases as the temperature increases. To further increase the temperature resistance of KGM, it is necessary to identify a novel method of improving the temperature resistance properties of KGM fracturing fluids.

In our research, an etherification modification of KGM (MKGM) was prepared. Chloroacetic acid was used as a modifier. The carboxyl group was introduced into the molecular structure under alkaline conditions. The branch chains on the etherification KGM molecular chains were extended, and the steric hindrance was increased, which would increase not only the attraction between molecules but also the probability of the extended branch chains intertwining with each other, making the intermolecular force greater. The etherification modification of KGM increased the viscosity.

This work examined the structure of modified KGM (MKGM) through ATR-FTIR and XRD. Both the organic titanium high-temperature stabilizer and the organic borate cross-linker were obtained to prepare MKGM and KGM gels. The thermal and rheological behaviors of the gels were tested using TGA and rheology measurements. The objective of our research was to evaluate the properties of MKGM and to make it more suitable for fracturing fluid.

## 2. Results and Discussion

### 2.1. ATR-FTIR Analysis

**Figure 2:**
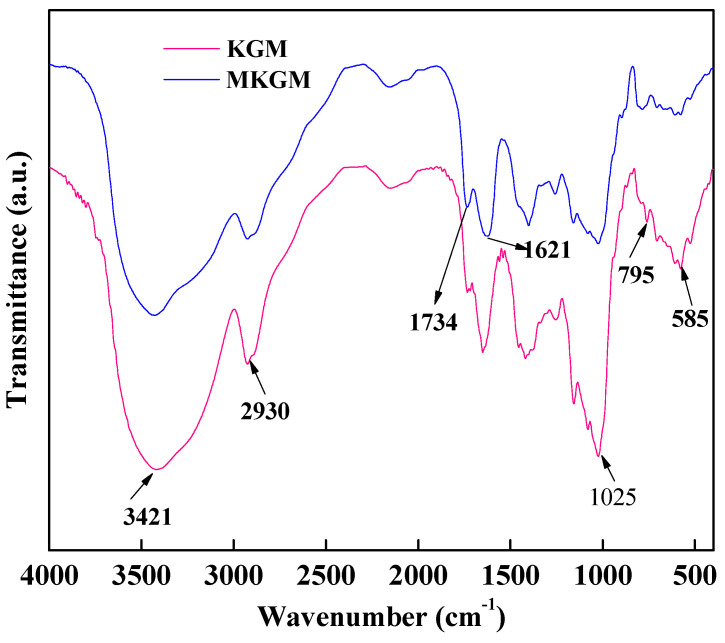
ATR-FTIR spectroscopy of KGM and MKGM.

The ATR-FTIR spectra of KGM and MKGM are shown in Figure 2. In the KGM spectrum, the broad absorption band at 3421 cm^−1^ corresponded to the stretching of hydroxyl. The band at 2930 cm^−1^ was due to the strong stretching modes of -CH_2_- and -CH-, which illustrated the existence of *O*-acetyl substitutions. The band at 1025 cm^−1^ was attributed to the C-O stretching vibrations. The bands from 795 to 585 cm^−1^ were due to the backbone ring of C-O-C [18].

Compared to KGM, the ATR-FTIR spectrum of MKGM at 873 and 807 cm^−1^ was not changed, which indicated that the backbone of the primary structure of MKGM was not changed during the modification reaction. The remarkable band at 1734 cm^−1^ was attributed to the carboxymethyl group, which showed that the etherification modification occurred. The band at 1621 cm^−1^ was due to the asymmetric absorption band of the carboxyl group. The results illustrated that MKGM was obtained through etherification modification.

### 2.2. XRD Analysis

**Figure 3:**
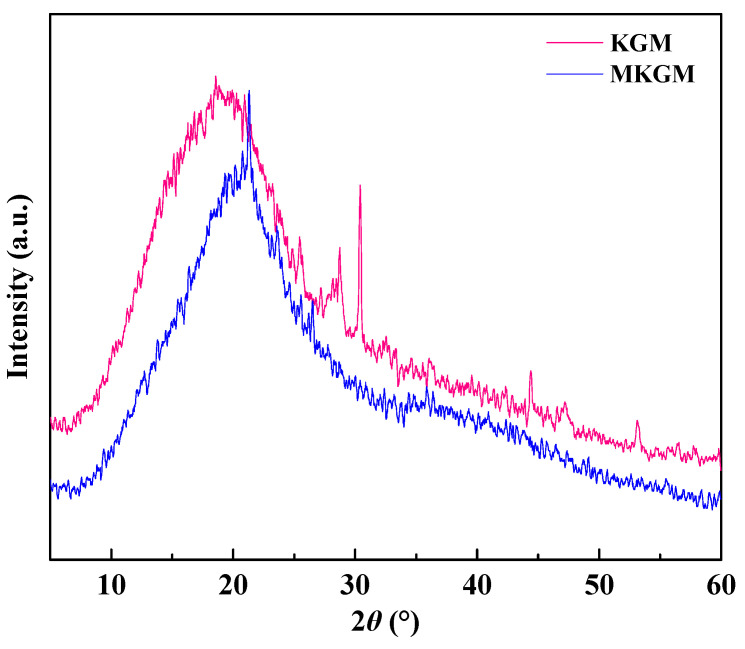
XRD patterns of KGM and MKGM.

XRD was used to probe the crystal behavior of the samples. The XRD patterns of KGM and MKGM are illustrated in Figure 3. The crystallinities of KGM and MKGM based on X-ray diffraction were 30.24% and 29.53%, respectively. The XRD pattern of KGM showed a broad peak at 2*θ* = 18.8° with two small and weak peaks appearing at 28.6 and 30.4°, which indicated that KGM was in the amorphous phase. The KGM pattern showed a broad pattern at 2*θ* = 21.2°, and the peaks at 28.6 and 30.4° were weakened [19,20]. The positions of the peaks shifted due to the low-angle diffraction. This showed that the crystallinity of MKGM decreased. Consequently, the crystal structure of KGM was proven to be destroyed due to the alkali and high-temperature modification conditions. Furthermore, the regularity of KGM was also destroyed after the introduction of the carboxymethyl group.

### 2.3. Thermal Analysis

#### 2.3.1. Thermal Stability Analysis

**Figure 4:**
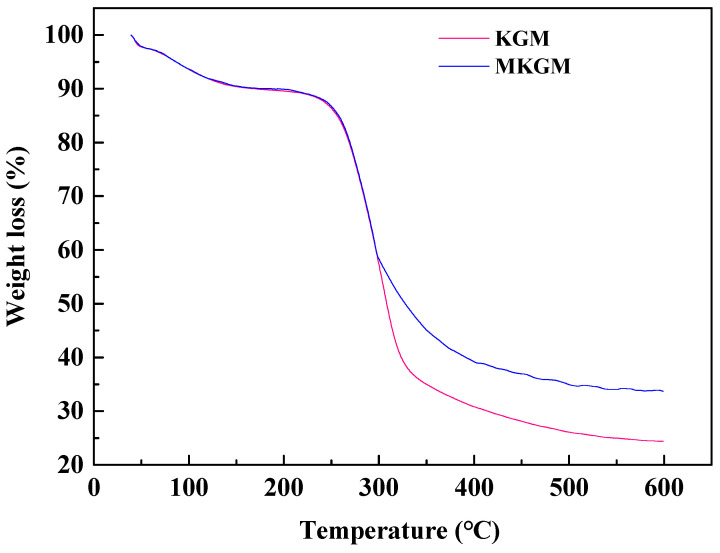
TGA curves of KGM(A) and MKGM(B).

The thermal gravimetric stability analysis of KGM and MKGM was investigated, and the results are shown in Figure 4. Both KGM and MKGM exhibited a three-stage thermal decomposition process including the low-boiling solvent loss stage, the molecular-chain-breaking loss stage, and the final thermal decomposition stage of coke. There is no obvious difference between KGM and MKGM since the temperature was below 100 °C. Both KGM and MKGM had a weight loss of 10% in the range of ambient temperature to 190 °C due to the evaporation of moisture from the samples. The major weight loss occurred as the temperature increased further from 190 to 420 °C, and an obvious weight loss step appeared after the first stage of loss of moisture at low temperatures. KGM began to decompose at 250 °C, rapidly losing 70% of its weight up to 325 °C, which could be attributed to the degradation of the backbone rings and the disintegration of the macromolecule chains of KGM. Beyond 325 °C, the weight loss was slow and gradual up to 600 °C, leaving about 25% residual weight. However, in the case of MKGM, the decomposition in the second step commences at 250 °C. With the introduction of the carboxymethyl group in MKGM, the difference in decomposition temperature appeared at 297 °C. This may be due to the decomposition of polymer chains carrying carboxymethyl groups. Beyond 400 °C, degradation proceeds at a very slow rate up to 600 °C. KGM showed a residual weight percent of 25% at 600 °C. However, the residual weight percent of 35% at 600 °C was higher than that of KGM, indicating the strong interactions of the carboxymethyl group in the molecular network. This result implied that the introduction of the carboxymethyl group into KGM was helpful in improving the thermal stability of KGM. These structural changes are bound to have different effects on the properties of gels. Thus, the thermal stability of MKGM was enhanced. The experiment was carried out under conditions similar to the previous reports, and the results were homologous [6].

#### 2.3.2. DSC Analysis

**Figure 5:**
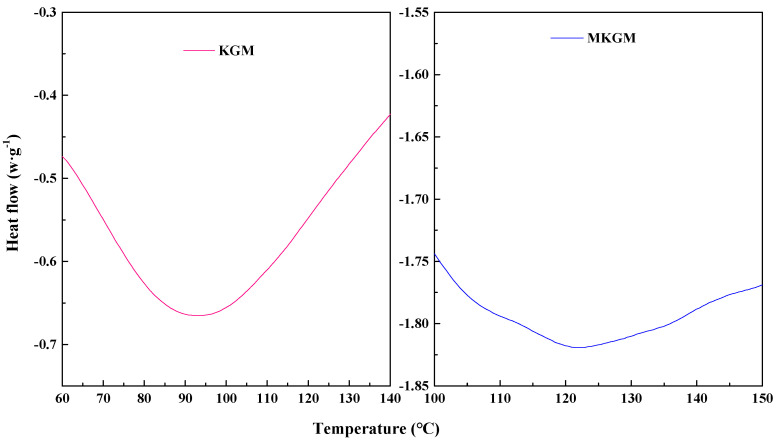
DSC curves of KGM and MKGM.

The DSC curves for KGM and MKGM are presented in Figure 5. It can be seen in Figure 5 that there were two broad endothermic peaks. The endothermic peak of KGM was 92.76 °C. This may be due to a loss of the hydroxyl group of KGM as water molecules. The KGM-H_2_O crystallite formed by hydrogen bonds was destroyed [14]. On the other hand, MKGM indicated a relatively broad endothermic peak around 121.13 °C, which was higher than that of KGM. The endothermic peak of MKGM increased after modification. This may be due to the introduction of the carboxymethyl group. The intermolecular force of MKGM increased and affected the thermal stability of KGM. The observed characteristics showed that the introduction of the carboxymethyl group has improved the thermal stability of KGM [21,22]. The result was consistent with the ATR-FTIR, XRD, and TG results.

### 2.4. Rheological Performances Analysis

#### 2.4.1. Temperature Resistance Test

**Figure 6:**
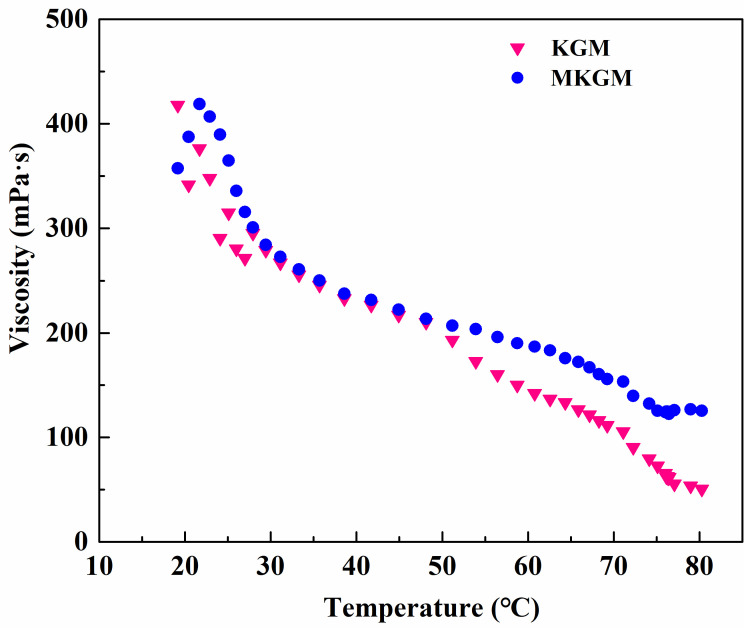
The effect of temperature on viscosity of KGM and MKGM gels.

The effect of temperature on the viscosity of the prepared KGM and MKGM gels under the same shear rate of 170 s^−1^ was investigated on a rheometer. The results are demonstrated in Figure 6. It can be seen that the viscosity of KGM and MKGM gels decreased as the temperature increased. The KGM and MKGM gels showed a clear thinning phenomenon. The increased temperature intensified the thermal movement of hydrophobic groups and water molecules in the KGM and MKGM structures, and then the viscosity of the KGM and MKGM gels decreased. However, the decreasing trend of the MKGM gel was relatively slower than that of the KGM. The viscosity of the MKGM gel was significantly higher than that of the KGM gel after 50 °C. This was due to the hydrophobic association in MKGM after modification. The hydrophobic association in MKGM was an endothermic-driven process. The intermolecular association may increase with increasing temperature. Thus, the viscosity of the MKGM gel tended to increase. Consequently, the viscosity of the KGM and MKGM gels generally decreased with the increase in system temperature, but the viscosity decreasing trend of KGM was higher than that of MKGM [23]. Moreover, the addition of titanium (IV) (triethanolaminato)isopropoxide also helped to enhance the thermal resistance. Titanium (IV) (triethanolaminato)isopropoxide was a chelate. The metal ion Ti^4+^ in the structure was the main element that could crosslink with the KGM and MKGM gels. Only a part of Ti^4+^ dissociated at the beginning to play a cross-linking role. When this part of cross-linked ions was heated, the balance was destroyed, and they could not fully combine with KGM and MKGM. The dissociated Ti^4+^ would be filled up to restore the balance and maintain the cross-linking effect, and so on until all chelate ions were completely dissociated. The test showed that MKGM had a better temperature resistance than KGM, which made it more suitable for fracturing fluid systems.

#### 2.4.2. Shear Resistance Test

**Figure 7:**
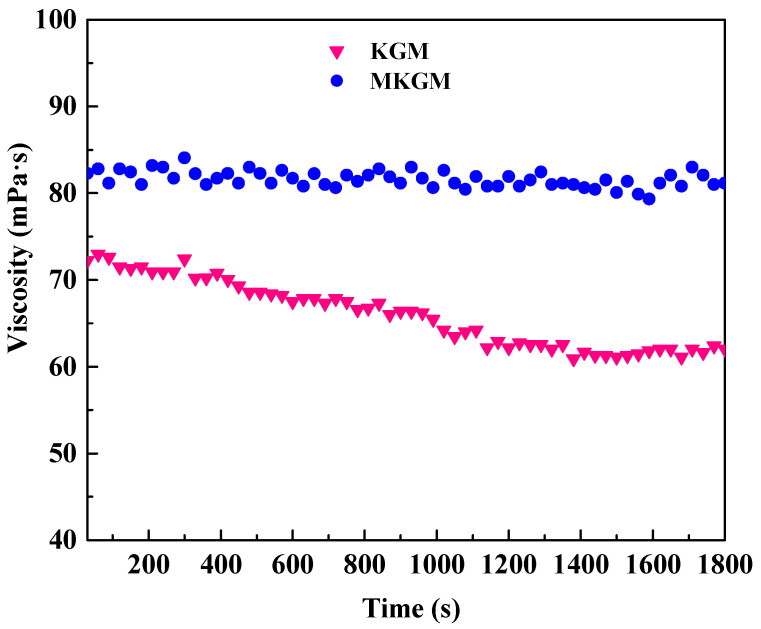
The shear resistance performances of KGM and MKGM gels.

The shear resistances of the prepared KGM and MKGM gels were investigated under steady shear conditions. Figure 7 shows the apparent viscosity versus time at a shear rate of 170 s^−1^, and a temperature of 80 °C for the KGM and MKGM gels.

The viscosity of the KGM gel decreased significantly compared to that of the MKGM as the time increased, as shown in Figure 7. However, the viscosity of the MKGM gel remained at a high value during the testing period [24]. The viscosity of the MKGM gel was less affected by shearing, showing that the MKGM gel had a better shear resistance. When the carboxymethyl group was introduced into the polymer chain, the hydrogen bond could be formed by the carboxymethyl groups. The entanglement of the hydrogen bond played a role in knots and formed a temporary three-dimensional network. The MKGM gel behaved as an elastic solid. Therefore, MKGM had a better shear resistance property, which would make it available for field fracturing applications.

#### 2.4.3. Viscoelastic Properties Test

The viscoelastic properties were a more characteristic index for evaluating the elasticity and viscosity of the gels. The elastic modulus (*G’*) and the viscous modulus (*G″*) were usually used to perform the viscoelastic properties [25]. In rheology, the relative value of *G″* to *G’* could reflect the viscoelastic properties. When the value of *G″* was lower than *G’*, the elastic modulus was higher than the viscous modulus. The elasticity was dominant, and the system was displayed as a solid feature, such as a gel state. When the value of *G″* was higher than *G’*, the elastic modulus was less than the viscous modulus. The viscosity was dominant, and the system was displayed as fluid features.

**Figure 8:**
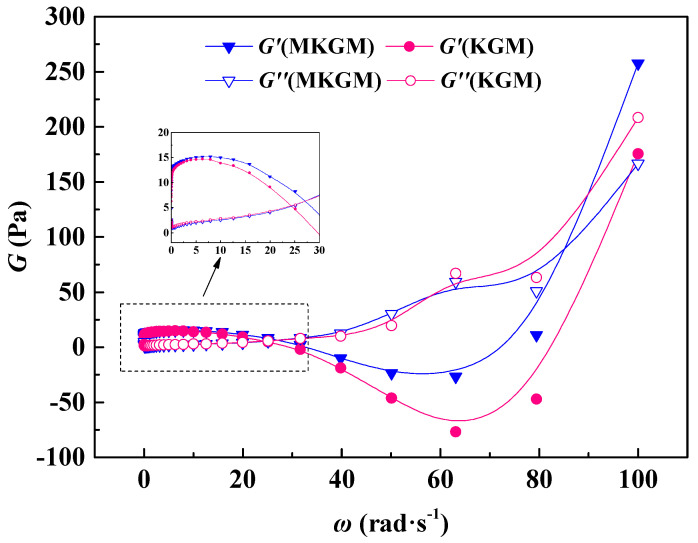
The variation of elastic modulus and the viscous modulus with frequency.

Figure 8 depicts changes in elastic modulus (*G’*) and viscous modulus (*G″*) as a function of angular frequency (*w*) for KGM and MKGM gels at 80 °C. It was noted that the magnitudes of *G’* and *G″* increased with increasing angular frequency for the KGM and MKGM gels.

When the frequency increased to 30 rad·s^−1^, the curves of the elastic modulus *G’* and viscous modulus (*G″*) approached, then crossed over each other, and the values of *w* at the intersected point of *G’* and *G″* were 23.92 and 27.28 rad·s^−1^, respectively. The *G’* values of the KGM and MKGM gels were higher than their *G″* values. It was proven that the elastic modulus of the gel system was higher than the viscous modulus in the dynamic modulus, whereas the system behaved mainly as an elastic fluid. The *G’* of MKGM was also higher than that of KGM because of the modification of KGM. This observable fact showed that MKGM displayed more elastic behavior at a lower frequency value. In general, the introduction of the carboxymethyl group into the polymer chain encourages the enhancement of viscosity, so the modified KGM possessed an elastic modulus value higher than that of the unmodified KGM. Although the frequency was above 30 rad·s^−1^, the *G″* values of KGM and MKGM were both higher than their *G’* values. The elastic modulus was lower than the viscous modulus in the dynamic modulus, and the system behaved mainly as a viscous fluid [26,27]. Therefore, it is suggested that the MKGM gel could be applied in fracturing fluid systems at a lower frequency. These results provide a theoretical understanding of the application of MKGM in improving sand-carrying capacity in fracturing and show that MKGM has great potential to be used in hydraulic fracturing.

## 3. Experimental

### 3.1. Materials

The raw KGM sample was purchased from Wuhan Qingjiang Konjac Products Co., Ltd., (Wuhan, China). Isopropanol (IPA), sodium hydroxide, chloroacetic acid, absolute ethanol, triethanolamine, borax, and sodium gluconate, analytically pure, were provided by Beijing Chemical Plant; butyl titanate was produced by Beijing East Chemical Co., Ltd., (Beijing, China); experimental water was deionized water. All other chemicals used in this study were laboratory-grade reagents. The organic titanium high-temperature stabilizer and the organic borate cross-linker were made in the laboratory.

### 3.2. Methods

#### 3.2.1. Preparation of Modified Konjac Glum (MKGM)

A certain amount of KGM was put into the three-necked glass flask equipped with a mechanical stirrer and a thermometer. Ethanol was added to the flask to remove impurities by stirring at room temperature for 2 h. The KGM powder was obtained by vacuum drying at 45 °C for 4 h.

**Figure 9:**
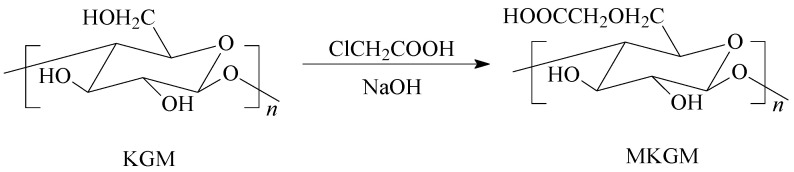
Synthesis of modified MKGM.

Amounts of 40 mL of IPA and 1 g of sodium hydroxide were added to the three-necked glass flask, and then the system was heated to 45 °C, stirring for 10 min [23]. An amount of 45 g of KGM powder was added to the solution system. An amount of 10 mL of chloroacetic acid was added to the solution when the KGM completely dissolved. Then, the modified KGM reaction occurred, and the reaction was allowed to proceed for 2 h. The MKGM was obtained by drying at 45 °C for 1 h after washing several times with absolute ethanol under vacuum filtration. The synthesis process of modified MKGM is demonstrated in Figure 9.

#### 3.2.2. Preparation of Organic Titanium High-Temperature Stabilizer

**Figure 10:**
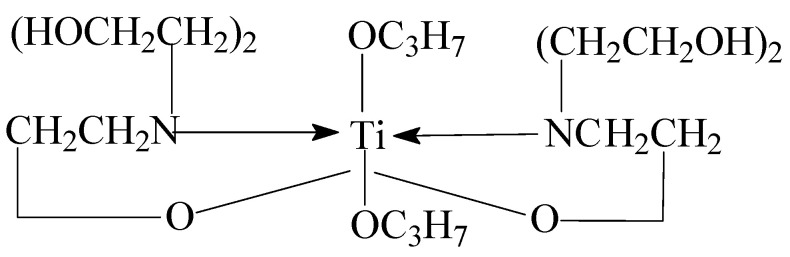
The structure of titanium (IV) (triethanolaminato)isopropoxide.

Triethanolamine was added to a three-necked glass flask under anhydrous conditions of 70 °C. Isopropanol was added under stirring conditions, and then butyl titanate was added after 1 h. A uniform transparent liquid organic titanium high-temperature stabilizer with a light yellow appearance was obtained via reaction at 70 °C for 3 h. The obtained stabilizer was a type of titanium chelate called titanium (IV) (triethanolaminato)isopropoxide. The structure of titanium (IV) (triethanolaminato)isopropoxide is shown in Figure 10.

#### 3.2.3. Preparation of Organic Borate Cross-Linker

A suitable amount of water was put into a three-necked glass flask, then borax, sodium gluconate, triethanolamine, and sodium hydroxide were added to the water one after the other at 70 °C under stirring over 4 h. Finally, the organic borate cross-linker was obtained.

#### 3.2.4. Preparation of the Fracturing Fluid System

(1) Preparation of MKGM and KGM base solution. A total of 0.35 g of MKGM was slowly added to 100 mL of water under vigorous stirring for 30 min after MKGM had uniformly dissolved in the water. The MKGM solution was then standing for 2 h before use. The KGM solution was also prepared following the above steps.
(2) Preparation of MKGM and KGM gels. Totals of 0.3 g of organic titanium high-temperature stabilizer and 0.8 g of organic borate cross-linker were both placed in MKGM base solutions (100 mL). A cross-linked MKGM gel was formed by manually stirring the mixture until the gel could be hung up easily. The KGM gel was also prepared following the above steps. Once the gels were prepared, they could immediately be used for evaluation tests [16].

### 3.3. Characterization

Accurately, 0.5 g of MKGM was added to a 250 mL conical flask equipped with a magnetic stirrer. An amount of 50 mL of 75% ethanol was added by stirring at 50 °C for 30 min. A saponification reaction started by adding 40 mL of 0.5 mol/L sodium hydroxide standard solution. The saponification reaction would last for 5 h. Afterward, three drops of phenolphthalein reagent were added. An amount of 0.5 mol/L hydrochloric acid standard solution was subsequently used to titrate until the red color had just disappeared. The volume of hydrochloric acid standard solution consumed was recorded as *V*_1_. A blank test was carried out with KGM. The volume of hydrochloric acid standard solution consumed was recorded as *V*_2_. The calculation equation of the degree of substitution (2-1, 2-2) was as follows:

(1) $$\omega \;(\%)=\frac{(V_{2}-V_{1})\times c\times 0.043}{m}\times 100\%$$

where *V*_1_ and *V*_2_ are the volumes of HCl used in MKGM and KGM during the titration, respectively; *c* is the concentration of HCl; and M is the weight of MKGM.

(2) $$DS=\frac{162\times \omega }{4300-\left(43-1\right)\times \omega }$$

where 43 is the relative molecular weight of acetyl; 162 is the relative molecular weight of each residue unit of KGM; and 1 is the relative atomic mass of the H atom.

The degree of substitution value of MKGM was 1.97 according to Equations (1) and (2).

The functional groups in KGM and MKGM were investigated by Bruker model V70 attenuated total reflection Fourier transform infrared (ATR-FTIR) spectroscopy in the range of 400–4000 cm^−1^ at a scan rate of 4 cm^−1^.

The X-ray diffraction (XRD) tests were performed with a Shimadzu XRD-6000 X-ray diffractometer using a CuKα radiation source (at the wavelength of 0.1542 nm) at room temperature, and the voltage and current were 40 kV and 40 mA, respectively. Patterns were recorded by monitoring diffractions from 5 to 60°. The scan rate was 0.02°/min. The degree of *crystallinity* of the samples was calculated using Equation (3) as follows:

(3) $$Crystallinity\;(\%)=\frac{A_{c}}{A_{c}+A_{a}}\times 100\%$$

where *A*_c_ and *A*_a_ are the areas of crystalline and non-crystalline regions, respectively.

Thermal gravimetric analysis (TGA) was performed on an American Q500 TG analyzer under a nitrogen atmosphere with a flow capacity of 20 mL/min. The sample was heated from ambient temperature to 600 °C at the ramping rate of 10 °C/min.

Differential scanning calorimetry (DSC) was performed on a Q200 differential scanning calorimeter (TA Instruments Company, USA). Samples were pretreated at 100 °C for 1 h to remove the absorbed water. The samples were then heated from 20 to 150 °C at a ramping rate of 10 °C/min under a nitrogen atmosphere.

The rheological properties of the gels were evaluated in an American TA Instruments AR2000ex Dynamic Rheometer. All rheological experiments were performed using a parallel plate geometry (40 mm in diameter and 1.5 mm in the gap). The samples were treated with constant shearing (1 s^−1^ for 10 min) before analysis to remove historical effects. The temperature resistance measurements were investigated at a temperature ranging from 20 to 80 °C at a shear rate of 170 s^−1^ after an equilibrium time of 120 s. The shear resistance measurements were investigated at a shear rate of 170 s^−1^ at 80 °C for 30 min. The viscoelastic property was studied at a constant temperature of 80 °C through a time sweep at various frequencies.

## 4. Conclusions

A new modification biopolymer (MKGM) was obtained using chloroacetic acid, IPA, and sodium hydroxide. The KGM and MKGM fracturing fluid systems were also prepared based on the organic titanium high-temperature stabilizer and organic borate cross-linker. Multiscale characterization methods were utilized to evaluate and compare the structure and performance of KGM and MKGM. ATR-FTIR results indicated that the carboxymethyl group was introduced into the KGM structure. The regularity of KGM was destroyed after modification by the XRD results. The results of TGA and DSC revealed that the thermal properties of the KGM were improved after modification. Furthermore, the rheological performance analysis demonstrated that the MKGM gel had better temperature and shear resistances than KGM. The MKGM gel displayed a more elastic behavior at a lower frequency value, and it could be applied in fracturing fluid systems at a lower frequency. In summary, MKGM had advantages over KGM. As a result, it is the method of modification that would best adapt the biopolymer to the field of hydraulic fracturing.

## Data Availability

All data generated or analyzed during this study are included in this article.

## Sample Availability

Samples of the compounds are not available from the authors.
